# Americans weigh an attended emotion more than Koreans in overall mood judgments

**DOI:** 10.1038/s41598-023-46723-7

**Published:** 2023-11-07

**Authors:** Gaeun Son, Hee Yeon Im, Daniel N. Albohn, Kestas Kveraga, Reginald B. Adams, Jisoo Sun, Sang Chul Chong

**Affiliations:** 1 Yonsei University, Seoul, South Korea; 2 University of British Columbia, Vancouver, Canada; 3 The Pennsylvania State University, University Park, USA; 4 Beth Israel Deaconess Medical Center, Boston, USA

**Keywords:** Psychology, Human behaviour

## Abstract

Face ensemble coding is the perceptual ability to create a quick and overall impression of a group of faces, triggering social and behavioral motivations towards other people (approaching friendly people or avoiding an angry mob). Cultural differences in this ability have been reported, such that Easterners are better at face ensemble coding than Westerners are. The underlying mechanism has been attributed to differences in processing styles, with Easterners allocating attention globally, and Westerners focusing on local parts. However, the remaining question is how such default attention mode is influenced by salient information during ensemble perception. We created visual displays that resembled a real-world social setting in which one individual in a crowd of different faces drew the viewer's attention while the viewer judged the overall emotion of the crowd. In each trial, one face in the crowd was highlighted by a salient cue, capturing spatial attention before the participants viewed the entire group. American participants’ judgment of group emotion more strongly weighed the attended individual face than Korean participants, suggesting a greater influence of local information on global perception. Our results showed that different attentional modes between cultural groups modulate social-emotional processing underlying people’s perceptions and attributions.

## Introduction

Face ensemble perception is the social cognitive ability to extract *gist* information from a crowd of faces^1,2^. Using this ability, our visual system rapidly extracts various summary statistics (e.g., mean, variance, or distributions) from a multitude of facial properties, including emotional expression^2–5^, identity^6,7^, gender^1^, race^8,9^, eye gaze direction^10^, and overall diversity or hierarchy in a facial group^11^. Such a perceptual means has been considered an effective strategy to achieve an accurate global representation of complex social environments with limited cognitive resources. However, given that any social beings interact with the world from their perspective of view, *perceiver effects*^12,13^, including social memberships and internal traits (e.g., anxiety and attentional state), must be incorporated for a deeper understanding of social ensemble perception.

A few studies have investigated this topic, especially regarding how an individual’s social relevance modulates the ability of facial ensembles^14–17^. Among them, recent studies focused on the impact of perceivers’ cultural background on ensemble perception. For example, Im and colleagues^4^ showed that the accuracy of extracting overall facial expressions from a group of people varied depending on their cultural background. When the participants from Korean and American cultures were presented with angry/happy crowd faces and were asked to decide which of the two crowds they would avoid, Korean participants chose the crowds with overall angrier emotions more accurately than American participants. Similar results were observed from Chinese and British participants^18,19^ such that Chinese participants were better at ensemble perception than British, suggesting that people from not just Korean but East Asian culture can perform social ensemble tasks better than the ones from Western culture.

A plausible mechanism underlying this cultural effect involves the different styles of perceptual processing of different cultural groups. In particular, previous work has proposed that people in Eastern and Western cultures tend to prioritize distinctive attention modes when processing visual information^20–25^. For example, Easterners tend to process information more holistically by distributing attention to contextual information surrounding a local target, whereas Westerners tend to focus on local information in an analytic way by dominantly attending to the target. This difference in information processing styles presumably originates from the cultural practice of “collectivism” vs. “individualism.” More specifically, in East Asian culture, people have historically prioritized an entire group (society) over individuals, so that the members of this society manifest a unique visual processing style that prioritizes the context. On the other hand, people in Western cultures have valued individuals over a group, which is indicated in their analytic perceptual processing style.

Such a view opens the possibility that one’s cultural background and associated information processing style can influence ensemble processing ability. According to both behavioral and modeling studies^26–28^, ensemble perception benefits from a distributed attention mode. When the attentional resource is distributed to multiple parts of visual field, the visual system can process those parts all together with no fine details, which is still enough to compose the accurate summary statistics. Peng et al.^18^ tested such a direct causal link between the different attentional modes of a cultural group and ensemble perception using a membership identification task. In this task, participants observed multiple faces in a single array and identified whether the subsequently displayed probe face was a member of the previous face array. If participants misidentify the average face of the array as a member in the previous array even though the average face had never been presented in the face array, it means the participants automatically represent the average face of the array. The result in Peng et al.^18^ showed that Chinese participants misidentified the average face more frequently than the British participants. More critically, when Chinese participants were using a selective attention mode immediately before performing this task, their misidentification of an average face decreased compared to when they were not using selective attention.

The remaining question, however, is how the default attention mode shaped by one’s cultural background operates in more dynamic socio-environmental settings. In realistic social interaction, two different modes of attention should work together to effectively extract relevant social information from a group of people. For example, to judge the intention of a crowd on the street accurately and efficiently, we should consider the facial expressions of its constituents but focus more on weighing the facial expression of its leader. A similar experimental scenario has been tested using non-social stimuli, under the framework of ‘weighted average’^29–33^. For example, visual stimuli were manipulated such that some parts of a scene attracted the participant’s attention via various attention cues. The human visual system was found to give weight to the cued stimulus; thus, the perceptual summary of stimuli was biased toward the attended local information. Furthermore, in the face domain, a recent study showed evidence of weighted averaging of facial expressions depending on the locus of attention^34^. This result suggests that attentional resources can be deployed adaptively even in a social context.

In the current study, we explored how the distinctive information processing styles across various cultural groups operate in cases where attention resources need to be flexibly allocated during ensemble perception. In other words, we tested how the weighted average mechanism is modulated by cultural differences during face ensemble perception, especially when local information *captured* viewers’ attention. To examine this, we conducted two experiments in which participants’ attention was attracted to one of the faces in a group using a strong exogenous attention cue before participants viewed the entire group of faces. Korean and American participants were recruited to represent the two distinct cultural groups of Easterners and Westerners, respectively. We investigated whether attentional cueing effects differentially impact the ensemble perception of a group of faces in these two cultural groups. Given that Westerners tend to utilize a more analytic perceptual processing style tuned to local information^20–22^, we hypothesized that American participants would be more strongly influenced by attention cues, showing a greater bias toward the attended local face during face ensemble perception. Conversely, we hypothesized that Korean participants would be less influenced by attention cues because of their greater tendency to leverage global information in general. To this end, we tested differential attentional cueing effects on ensemble perception originating from one’s culture in the following two experiments. This study was not preregistered.

## Experiment 1: Ensemble perception of East Asian faces

In Experiment 1, we compared the attentional cueing effect during emotion ensemble perception between Korean and American participants using stimulus arrays consisting of ‘Korean’ faces. The faces had either angry or happy facial expressions, and the ratio between the two facial expressions in the array varied by seven levels. We presented an exogenous attention cue to the location of one individual face among the array of multiple Korean faces before the participants viewed the entire face array and asked them to judge whether the overall mood of the array (i.e., the entire group of faces) was positive or negative. One thing to emphasize here is that we utilized an exogenous attention cue to maximize the effect of attention in a bottom-up manner. Sudden contrast increment of a cue and a short cue leading time ensured the capture of participants’ attention^35^. To summarize our results, we found that while averaging the mood of a group of Korean faces, American participants were more strongly influenced by the emotion of the cued face than Korean participants and thus showed less precise and more biased responses.

### Methods

#### Participants

A total of 107 participants were recruited for Experiment 1. Fifty participants were Korean undergraduate students from Yonsei University (25 women and 25 men, native Koreans with no living-abroad experiences more than one year) and the other 57 were American undergraduate students from Pennsylvania State University (30 women and 27 men, race information missing—a careful discussion is included in the Discussion section). All participants reported normal or corrected-normal vision and provided written informed consent before participation. Korean participants were paid 10,000 KRW (approximately 10 USD), and American participants were given a course credit of one hour. Among these participants, four Korean and 14 American participants were excluded from the final analysis (detailed exclusion criteria are described in the Analysis section). After exclusion, the data from 89 participants were included in the final analysis. This sample size was arbitrarily determined because we could not find any study that used a design similar to ours, but the achieved statistical power comparing the multilevel regression models with and without the culture variable was 100% [95% CI 88.43, 100.0]. This value was obtained by the SiMR package^36^ that simulates statistical power for multi-level regression models in R 4.1.2^37^. Notably, we used this power to estimate an adequate sample size in Experiment 2 and replicated the effects with an independent group of participants. This study was approved by the institutional review boards of Yonsei and Pennsylvania State Universities. All methods were performed in accordance with the relevant guidelines and regulations by the Institutional Review Boards.

#### Apparatus and stimuli

All stimuli were generated using MATLAB and the Psychophysics Toolbox^38^. To generate East Asian face arrays, we used the Yonsei Face Database^39^, which consists of seven facial expressions (fearful, happy, surprised, sad, disgusted, angry, and neutral) from 17 models (nine women and eight men). From this set, happy and angry facial expressions of eight women and eight men were selected for the experiment. The stimuli were cropped to an oval shape (1.72° × 2.24°) that retained the major facial components of the eyes, nose, and mouth. Given the selected faces, each array was created by randomly presenting eight individual faces with either happy or angry emotional expressions. The locations of the eight faces were prefixed by placeholders. Each placeholder was a gray oval ring of the same size as that of a cropped face (1.72° × 2.24°). These placeholders were positioned at eight equidistant points on an imaginary circle (radius of 3.37°) from the center fixation cross (arm length: 0.12°). All faces were presented as grayscale images.

#### Design

We used a 2 × 3 × 7 mixed design with one between-subject factor and two within-subject factors. The between-subjects factor was the participants’ cultural group, with two levels: Korean and American. The first within-subject factor was the type of attention cue, with three levels: angry face cues (AC), happy face cues (HC), and no cues (NC). Specifically, in the AC and HC conditions, we presented a high-contrast attention cue very briefly at the location of the subsequent angry and happy faces, respectively, to attract the participants’ attention to the specific individual face. In the NC condition, no attention cue was presented, serving as a baseline for comparison with the performance in the AC and HC conditions. The second within-subject factor was the positive valence ratio with seven levels. We varied the ratio between happy and angry faces at seven levels: 1:7, 2:6, 3:5, 4:4, 5:3, 6:2, and 7:1. Importantly, while the overall facial expressions varied, we maintained a constant ratio of female and male faces to control the potential interaction of gender and facial expression^40,41^. When the number of happy faces was even (e.g., 2:6, 4:4, 6:2), the face crowds always consisted of the same number of happy women and men. For example, in the 2:6 ratio of happy to angry, there is one happy woman, one happy man, three angry women, and three angry men in the crowd. For half of the trials in which the number of happy faces in the crowd was odd (e.g., 1:7, 3:5, 5:3, 7:1), one more happy female face was included in the stimulus, whereas the other half contained one more happy male face than the female. Based on this design, participants completed 504 trials in total (3 types of attention cue × 7 levels of valence ratio with 24 repetitions).

#### Procedure

**Figure 1:**
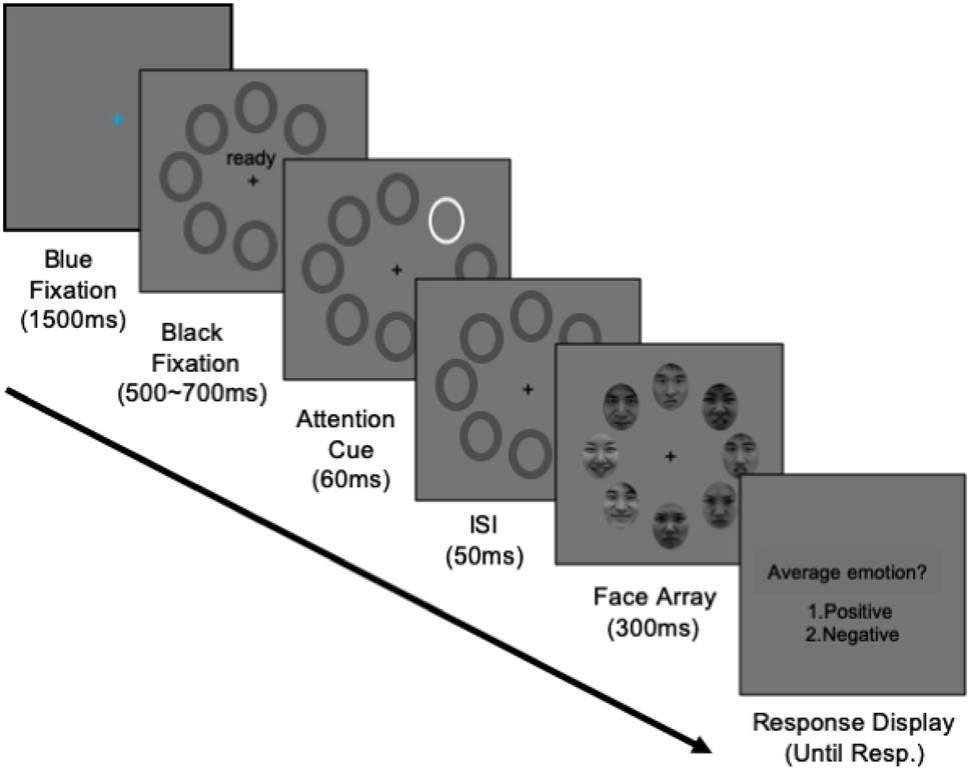
Experiment procedure. Example trial of Experiment 1. For the Korean participants, instructions on the black fixation display and response display were written in Korean.

An example of a trial sequence is shown in Fig. 1. Each trial began with a blue fixation cross, which turned into a black fixation in 1500 ms. When the fixation cross changed its color, a “ready” sign appeared above the fixation, along with eight placeholders to inform participants of the upcoming presentation of a face array (jittered SOAs: 500–700 ms). After the fixation cross disappeared, a high-contrast attention cue (a white oval frame) was presented at one of the eight placeholders for 60 ms in the AC and HC conditions. No attention cues were presented in the NC condition. After the cue disappeared, a black fixation cross with placeholders reappeared for 50 ms, followed by the presentation of a group of eight faces with different emotional expressions for 300 ms. Such a short interval between the cue and face displays ensured that participants cannot redirect their attention away from the cued location. After the face array disappeared, participants were asked to judge the overall emotional valence (negative vs. positive) of the face array by pressing the matching keys. The participants were instructed to fixate on the fixation cross during the entire trial, except in the blue fixation period. This instruction prevented participants from making explicit eye movements to the cued faces and responding solely based on the cued face rather than an array of eight faces. No feedback was provided, except in the 15 practice trials in a separate block. In the practice trials, high-pitched brief audio sound was provided only after the incorrect responses.

#### Analysis

##### Data exclusion criteria

In the current experimental design, where the number of happy faces continuously increased in seven steps, the probability of judging a certain face array as “positive” should also increase correspondingly, although the speed or initial point of the linear increment varied across attention cue conditions. If we could not observe such a trend, it was assumed that the participant did not correctly understand the task. Therefore, we conducted initial logistic regressions for each attention cue condition on individual participants’ data using R 4.1.2^37^. In the model, we predicted participants’ responses using the positive valence ratio as a predictor. If the coefficient of this predictor did not reach a significant level in any of the three attention cue conditions, it could be inferred that the probability of “positive” responses did not increase with the positive valence ratio, suggesting that the participants did not perform the task appropriately. Based on this criterion, we excluded 18 participants (four Koreans and 14 Americans) from the final analysis; therefore, the total number of participants included in the final analysis was 46 Koreans and 43 Americans in Experiment 1. Supplementary Figs. 1 and 2 show the performance of the individual participants.

##### Multi-level logistic regression

After data exclusion, multilevel logistic regression was applied to the final dataset. The regression model was designed to predict the probability of “positive” responses (*p*(“positive response”)) with a positive valence ratio, attention cue, culture, and all possible interactions between these variables. In the model, we included a random slope of participants because we observed substantial slope variability in the AC and HC conditions across participants (see Supplementary Figs. 1 and 2). This was estimated with an unstructured covariance matrix using the ‘glmer’ function with a logit link function in the LME4 package^42^ in R 4.1.2^37^. To correctly estimate the significance of the categorical predictor with more than two levels (attention cues in our design), we conducted a chi-square analysis that compared pairs of nested models with and without a predictor (or an interaction). This approach tested whether including an additional predictor or interaction term to a model increased the explanatory power of the model. According to our hypothesis that American participants weigh the attended face more than other faces during ensemble perception, we anticipated a significant interaction between attention cue and culture, or a three-way interaction with positive valence ratio. The two-way interaction was expected when the overall effect of the attention cues was modulated by culture. On the other hand, a significant three-way interaction was expected when the slopes predicting *p*(“positive response”) as a function of the positive valence ratio were modulated by the attention cues while the size of modulation varied across cultures.

To identify the exact sources of significant interactions, we additionally conducted a post hoc pairwise comparison with Tukey adjustment using the ‘emmeans’ package^43^. For a significant two-way interaction, we compared the marginal means of AC (or HC) to that of the baseline NC separately for the two cultural groups. For a significant three-way interaction, we compared the slopes of AC (or HC) predicting the *p*(“positive response”) as a function of the positive valence ratio to the slope of the baseline NC separately for the American and Korean groups.

##### Decision boundaries and regression slopes

After checking the overall effects of attention cues and culture on ensemble perception in the group-level regression, we calculated regression slopes and decision boundaries at the individual participant level. This additional analysis allowed us to further examine whether the differential attention cueing effect between the two cultural groups is based on task precision, bias, or both. A regression slope is interpreted as the precision with which the participants judge the average emotion of a face array. Specifically, a steeper slope indicates that participants can detect a small increment in happy faces in a group with higher sensitivity. The decision boundary reflects performance bias. This was calculated by shifting the predicted point *p* (“positive responses”) from 0.5, along with the scale of the positive valence ratio. With no bias, the decision boundary should be located at the midpoint of the positive valence ratio (happy: angry = 4:4). If a decision boundary is located below 4:4, then we can infer that a participant judges a face array “positive” more liberally with fewer happy faces in the array. On the other hand, if a decision boundary is located above 4:4, participants need more happy faces to judge an array as “positive.” To statistically evaluate such patterns of precision or bias across different attention cues and cultures, we conducted a multi-level linear regression that predicted the precision (or bias) with varying attention cues and cultures using a random intercept of participants. Then, we conducted analysis of variance (ANOVA) to compare pairs of nested models and a subsequent post hoc pairwise comparison using the ‘emmeans’^43^ package in R for any significant interactions.

### Results

**Figure 2:**
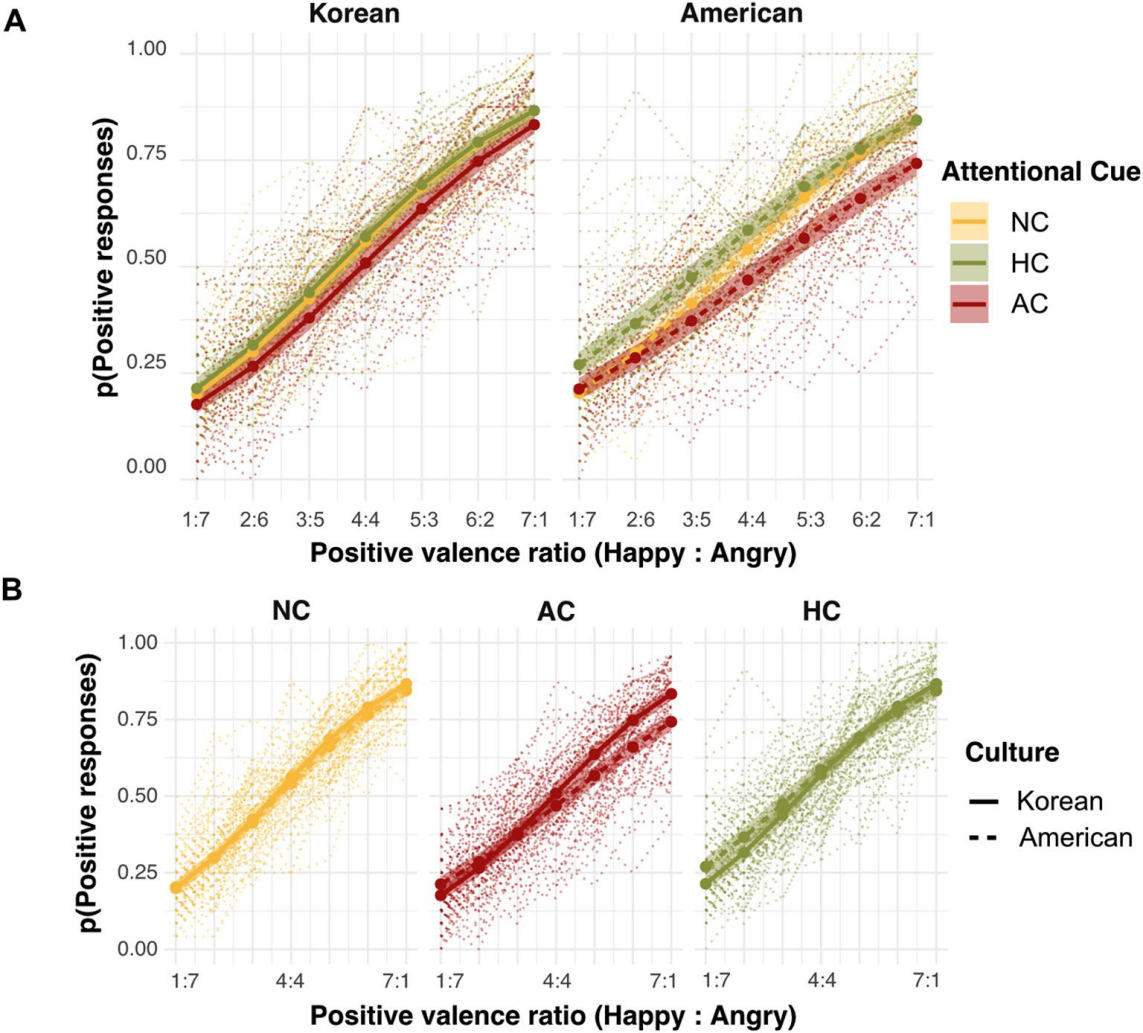
Results of Experiment 1. The thick solid (Korean) and dashed (American) lines indicate the predicted probability of a positive response using the regression model. Shaded areas represent the 95% confidence interval and dotted lines indicate individual participants. AC, attention cue on angry faces; HC, attention cue on happy faces; NC, no attention cue (baseline). (**A**) Overall response patterns of the two cultural groups. The lines for the three attention cue conditions overlap less for Americans, implying stronger attentional cueing effects than for Koreans. (**B**) A more direct comparison of cueing effects between the two cultural groups for each cue condition. In the NC condition, the two cultural groups showed comparable performance. In the AC condition, Americans judged the average emotion as less positive when the actual positive valence ratio was higher (right). In the HC condition, Americans judged the average emotion to be more positive when the positive valence ratio was lower (left).

Table 1 presents the full list of effects in Experiment 1. As the positive valence ratio increased, both participant groups responded “positive” with a higher probability (a main effect of positive valence ratio: χ^2^(1) = 1423.02, *p* < 0.001), suggesting that the participants properly evaluated the overall mood of the faces along the different levels of positive valence ratio. In addition, the probability of “positive” responses was affected by the attention cue (a main effect of attention cue: χ^2^(1) = 13.58, *p* = 0.001), indicating an attentional cueing effect on face ensemble perception. Importantly, this cueing effect was modulated by cultural group and positive valence ratio (a significant three-way interaction between culture, attention cue, and positive valence ratio: χ^2^(2) = 11.90, *p* = 0.003, see Fig. 2A). In the subsequent post hoc comparisons, we observed that this interaction was mainly driven by American participants, especially as reflected in the shallower slopes of the AC (adjusted *p* < 0.001) and HC (adjusted *p* = 0.005) than the NC. No slope differences were observed in the Korean participants (adjusted *p*s > 0.65).

**Table 1:** Experiment 1 multi-level model results.

Effects	χ^2^
Positive valence ratio	1423.02***
Attention cue	13.58**
Culture	1.16
Positive valence ratio × attention cue	0.87
Positive valence ratio × culture	2.59
Attention cue × culture	3.77
Positive valence ratio × attention cue × culture	11.90**

***p < 0.001, **p < 0.01, *p < 0.05.

We scrutinized the differential effects of the AC and HC conditions by calculating the precision (individual regression slopes) and bias (decision boundaries) of participants’ responses. When comparing the *precision* of ensemble perception, we observed a significant interaction between attention cue and culture (χ^2^(2) = 13.42, *p* = 0.001). This interaction was mainly driven by the American participants’ less-precise ensemble performance in both the AC (adjusted *p* < 0.001) and HC (adjusted *p* = 0.003) than in the baseline NC. Such a precision difference between the attention cues was not observed in the Korean participants (adjusted *p*s > 0.62).

**Figure 3:**
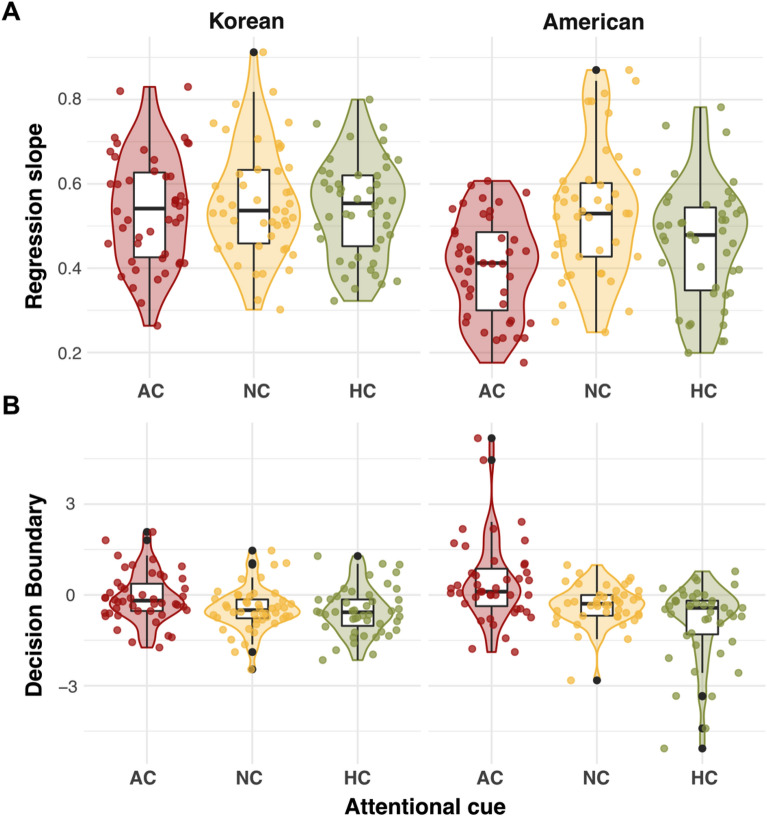
Individual participants’ regression slopes and decision boundaries. (**A**) Regression slope. The lower slopes in American participants, especially in both the AC and HC conditions, show that they performed the ensemble task less precisely, without considering the increment of the positive valence ratio in a sensitive manner. (**B**) Decision boundary. Only American participants showed noticeable biases (deviated from 0) in the AC and HC conditions in the opposite direction, showing response biases toward cued faces.

Furthermore, when comparing the *decision bias* (Fig. 3B) in ensemble perception, we again observed a significant interaction between attention cue and culture (χ^2^(2) = 8.77, *p* = 0.01). This interaction was driven by significant decision bias in the AC (adjusted *p* = 0.001) and HC (adjusted *p* = 0.03), especially among the American participants. Specifically, in the AC condition, the decision boundary in American participants was above zero, suggesting that American participants were strongly influenced by the cued angry face during ensemble perception; thus, they needed to see more happy faces to judge the facial crowd as positive. For the HC condition, however, the decision boundary in American participants was below zero, suggesting that they judged a facial crowd as positive, even with fewer happy faces in a crowd as affected by the cued happy face. The Korean participants showed no significant bias in either condition when compared to the baseline NC (adjusted *p*s > 0.14), as shown by very weak bias (around 0) across all three attention cue conditions. In sum, the stronger response bias and shallower slope observed from American participants in the AC/HC conditions suggest that they relied more on cued individual faces during ensemble face perception (Table 2).

**Table 2:** Experiment 1 precision and bias analysis.

Effects	χ^2^
Precision (regression slopes)	
Attention cue	1.05
Culture	1.00
Attention cue × culture	13.42**
Bias (decision boundary)	
Attention cue	5.98
Culture	0.43
Attention cue × culture	8.76*

***p < 0.001, **p < 0.01, *p < 0.05.

## Experiment 2: Ensemble perception of Caucasian faces

In Experiment 1, we observed that American participants were more strongly influenced by the emotion of an individual face, which captured selective attention when perceiving the average emotion of a group of faces. However, this finding could be restricted to the type of facial stimuli used in the experiment. Specifically, in Experiment 1, the race of the face stimuli was Korean, which benefits Korean participants due to the high familiarity. To rule out this possibility, we generalized the effects to the Caucasian faces that would be more familiar to American participants. In Experiment 2, we questioned whether American participants would still be affected more strongly by attention cues than Koreans when face arrays were composed of Caucasian faces that they would be more familiar with and presumably process better. To briefly overview the results, we replicated the stronger cueing effect in American participants than in Koreans using Caucasian face arrays.

### Participants

A new cohort of 100 undergraduate students participated in Experiment 2. Fifty participants were Korean undergraduates at Yonsei University (27 women and 23 men, native Koreans with no international experiences longer than 1 year), and the other 50 were American undergraduates at Pennsylvania State University (23 women and 27 men, race information missing). Three Korean and five American participants were excluded from the final analysis, based on the same exclusion criteria as in Experiment 1 (Supplementary Figs. 3 and 4). This sample size was predetermined based on the power simulation method in the SiMR package^36^ in R 4.1.2^37^ using the data collected in Experiment 1. Given the effect size observed in Experiment 1, we simulated statistical power by increasing the number of participants. In the simulation, we could achieve a power of 90% using 52 participants. Thus, we decided to collect data from a similar number of participants to that in Experiment 1. All participants reported having normal or corrected-to-normal vision and provided written informed consent. For their participation, Koreans received monetary rewards (10,000 KRW, approximately 10 USD) and Americans received course credit.

### Apparatus, stimuli, procedure, and analysis

The overall methods of Experiment 2 were the same as those in Experiment 1, except for the race of the face stimuli. To compose Caucasian face arrays, we used the FACES database^44^ containing six facial expressions (neutral, sad, disgusted, fearful, angry, and happy) of 171 models (58 young, 56 middle-aged, and 57 older men and women). Because the models of the Yonsei Face Database were relatively young (mean age = 24.71 years, SD = 3.87, age range: 20–31), we used the young group models of the FACES database (mean age = 24.3 years, SD = 3.5, age range: 19–31). From this stimulus set, we selected happy and angry facial expressions of 16 identities (eight women and eight men). These stimuli were also cropped to have the same oval shapes as the Korean face stimuli and used to generate face arrays at seven different levels of emotional valence. The task procedure and analyses were the same as those in Experiment 1.

### Results

**Figure 4:**
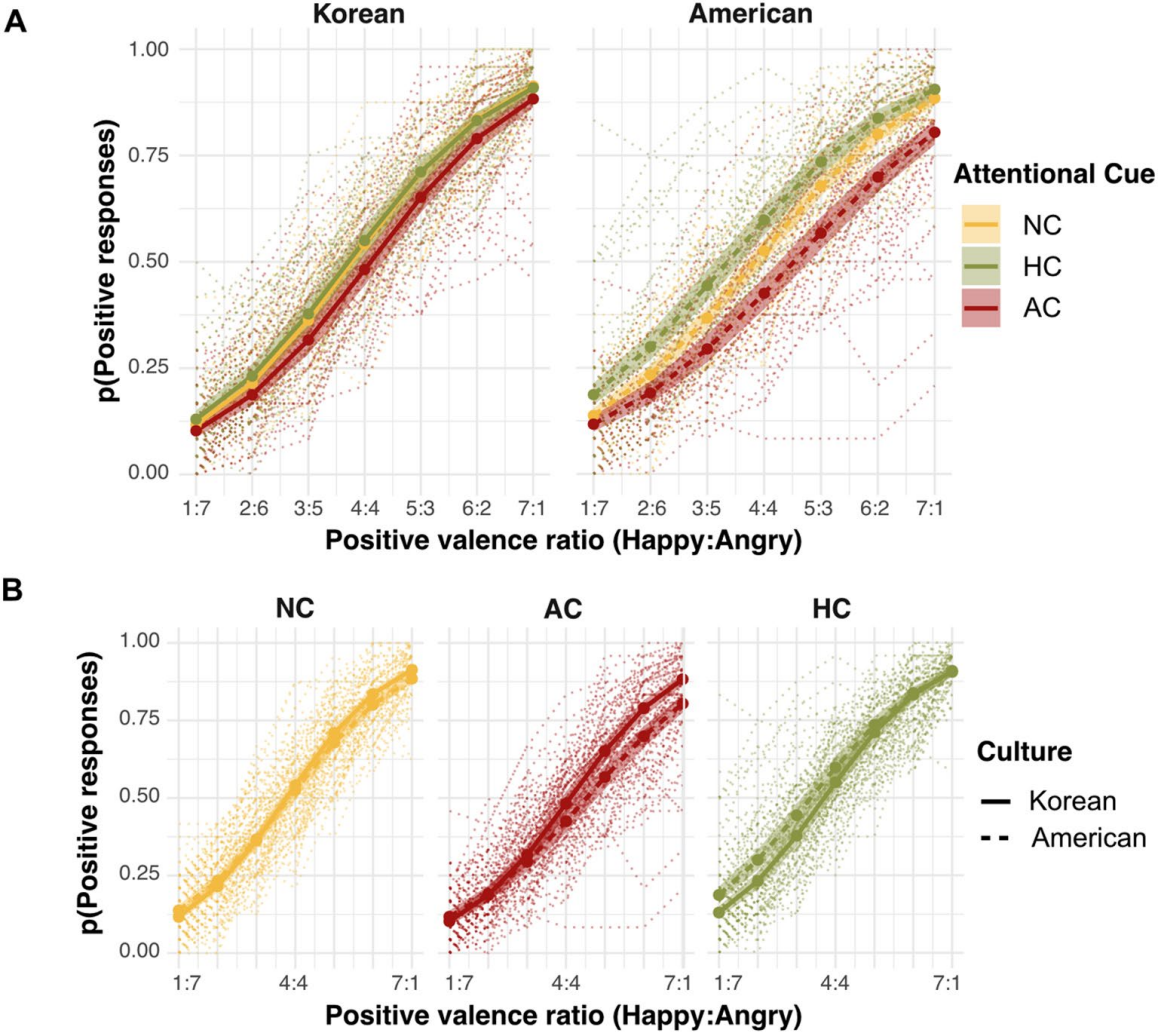
Results of Experiment 2. Shaded areas are the 95% confidence intervals, and dotted lines indicate individual participants. AC, attention cue on angry faces; HC, attention cue on happy faces; NC, no attention cue (baseline). The results were highly similar to those of Experiment 1, in which the face array was composed of Korean faces.

Table 3 presents the full list of observed effects in Experiment 2. We again observed the main effects of positive valence ratio (χ^2^(1) = 1973.61, *p* < 0.001) and attention cue (χ^2^(1) = 13.55, *p* = 0.001), which were consistent with the results of Experiment 1. While we observed a significant three-way interaction in addition to these main effects in Experiment 1, we did not observe the same three-way interaction in Experiment 2 (χ^2^(2) = 2.76, *p* = 0.25). Instead, we observed significant interactions between positive valence ratio and culture (χ^2^(1) = 13.71, *p* < 0.001) and between attention cue and culture (χ^2^(2) = 7.01, *p* = 0.03). However, the second interaction suggested differential influences of the attention cues between the Korean and American participants (Fig. 4). Furthermore, the overall *p*(“positive response”) was still modulated by different attention cues, although the slopes predicting positive responses as a function of the positive valence ratio did not differ. In the subsequent post hoc comparison, we observed that especially in the American participant group, ensemble performance was modulated by both the AC (adjusted *p* < 0.001) and the HC (adjusted *p* < 0.001). This time, Korean participants’ ensemble performance was also modulated by the AC (adjusted *p* = 0.002).

**Table 3:** Experiment 2 multi-level logistic regression results.

Effects	χ^2^
Positive valence ratio	1973.61***
Attention cue	13.55**
Culture	0.76
Positive valence ratio × attention cue	2.32
Positive valence ratio × culture	13.71***
Attention cue × culture	7.01*
Positive valence ratio × attention cue × culture	2.76

***p < 0.001. **p < 0.01. *p < 0.05.

**Figure 5:**
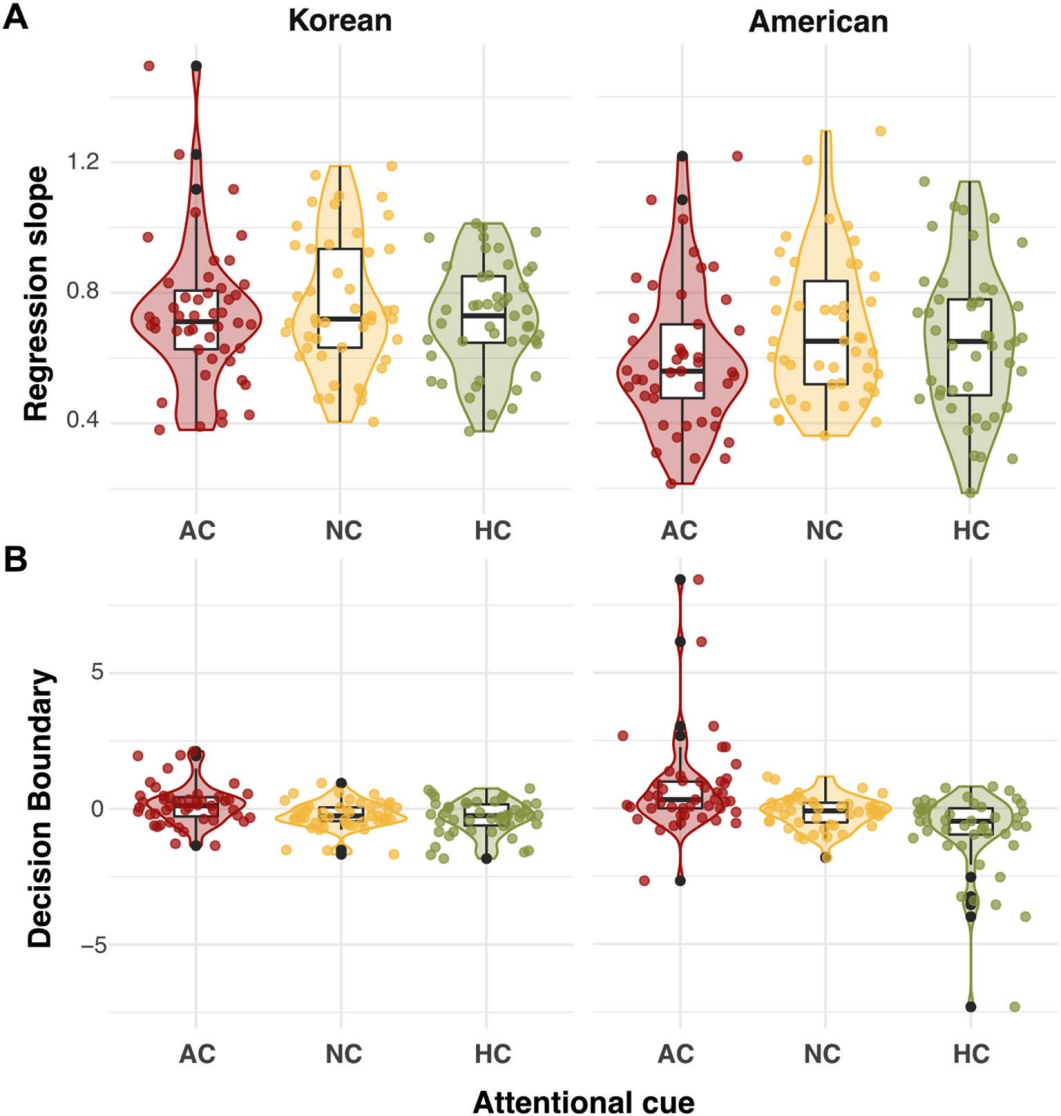
Regression slopes and decision boundaries in Experiment 2. (**A**) Regression slope. The lower slopes in American participants across all conditions showed that they performed the ensemble task less precisely in general, even with familiar Caucasian faces. In this study, we did not observe lower slopes from the AC/HC conditions in Americans. (**B**) Decision boundaries. Only American participants showed noticeable biases (deviated from 0) in the AC and HC conditions in the opposite direction, showing response biases toward cued faces.

We further scrutinized the cueing effects analyzing precision (regression slopes) and bias (decision boundary) (Table 4). Regarding precision, we found overall lower ensemble precision in the American participants than the Korean participants, suggesting that Americans were less sensitive in perceiving the increment of the actual positive valence ratio in their ensemble judgments than the Korean participants (a main effect of culture: χ^2^(1) = 6.72, *p* = 0.009). We also found significant cueing effects when the cultural groups were aggregated (a main effect of attention cues: χ^2^(2) = 9.05, *p* = 0.01). However, the interaction between culture and the attentional cueing effect did not reach a significant level (χ^2^(2) = 2.88, *p* = 0.24), suggesting that the differences in ensemble precision induced by different attention cues were comparable between the two culture groups.

**Table 4:** Experiment 2 precision and bias analysis.

Effect	χ^2^
Precision (regression slopes)	
Attention cue	9.05*
Culture	6.73**
Attention cue × culture	2.88
Bias (decision boundary)	
Attention cue	43.16***
Culture	0.14
Attention cue × culture	12.68**

***p < 0.001. **p < 0.01. *p < 0.05.

Regarding bias, we observed a significant interaction between attention cue and culture (χ^2^(2) = 12.78, *p* = 0.001). This interaction was driven by significant decision bias in the AC (adjusted *p* = 0.003) and HC (adjusted *p* = 0.007). More specifically, the decision boundary for the AC condition was above zero for American participants, suggesting that they needed to see more happy faces to judge the facial crowd as positive. The decision boundary for the HC condition was below zero for American participants, suggesting that they tended to judge the facial crowd as positive with fewer happy faces. No such pattern was observed in the Korean participants (adjusted *p*s > 0.17), who showed very weak bias (around zero) across the attention cue conditions. In sum, these results reveal a clear pattern of stronger cueing effects in Americans than Koreans. However, different from Experiment 1, this effect was mostly driven by decision bias rather than ensemble precision. In the following section, we directly compared the ensemble precision and decision boundary between Experiments 1 and 2 to evaluate any modulation effects that were more directly related to the race of the facial stimuli.

## Cueing effect comparison between Experiments 1 and 2

**Figure 6:**
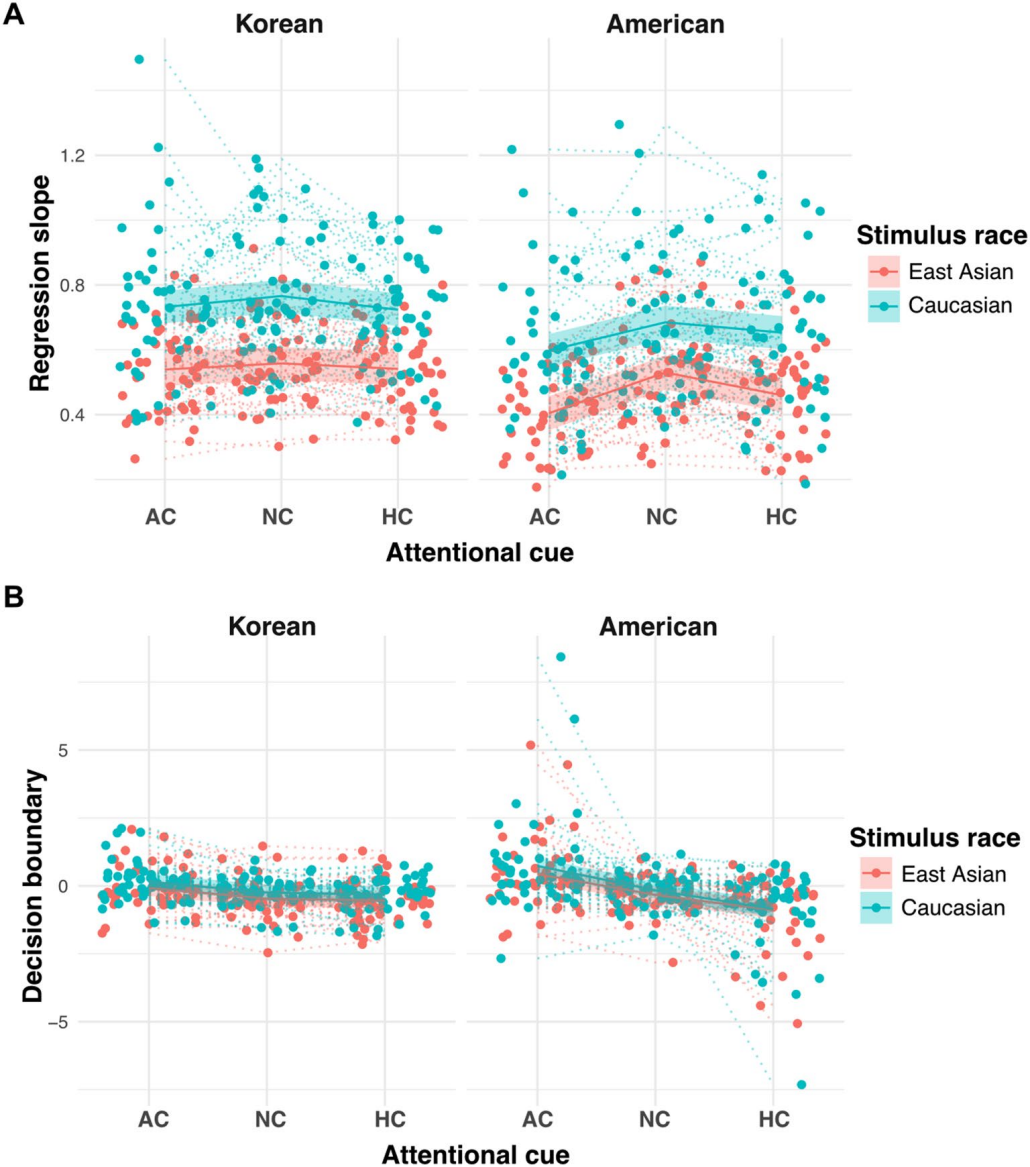
Cueing effect comparison between two experiments. Figures 3 and 5 are compared in a different style for easier analysis. The jittered dots and dotted lines indicate the individual participants’ data. The solid line surrounded by the shade indicates group-level prediction lines with CI 95%. (**A**) Regression slope comparison. In general, the slopes were lower in the American participants than in the Korean participants. However, for American participants, the slopes were particularly lower in the AC/HC condition than in the NC condition. (**B**) Decision boundary comparison. For the Korean participants, decision boundaries were clustered around 0 across all attention cue conditions, regardless of the stimulus race. However, American participants showed higher decision boundaries in the AC condition and lower boundaries in the HC condition than in the NC condition. This trend remained the same regardless of the stimulus race.

While the observed cueing effect in two Experiments pointed the same conclusion such as the stronger cueing effect in Americans than Koreans, there were small differences in the statistical results supporting this conclusion. In multi-level regression, we observed the modulation effect of culture on attention from the 3-way interaction including the valence ratio in Experiment 1 but from the 2-way interaction in Experiment 2. Also, in the precision/bias analysis, we identified the culture-attention interaction from both precision and bias values in Experiment 1, but only from the bias value in Experiment 2. To directly compare the cueing effects between the two experiments, we conducted a multi-level regression analysis that predicted either precision (regression slopes) or bias (decision boundaries) with the attention cue, culture, stimulus race, and interaction terms among these variables with random intercepts for individual participants. A full list of effects is displayed in Table 5. We expected that if each cultural group showed different cueing effects depending on the race of the face stimuli, we would observe a significant 3-way interaction among attention cue, culture, and stimulus race. In the precision model (Fig. 6A), we did not observe this interaction (χ^2^(2) = 1.84, *p* = 0.40) or any other stimulus race-driven interactions (*ps* > 0.75). However, we found a significant main effect of culture (χ^2^(1) = 13.14, *p* < 0.001), revealing that Korean participants were, in general, more precise in their judgments of ensemble tasks. We also observed a significant main effect of stimulus race (χ^2^(1) = 28.10, *p* < 0.001), indicating that the ensemble task with Caucasian faces was generally more precise than that with Korean faces. In addition, an interaction between culture and attention cues was also significant (χ^2^(2) = 10.90, *p* = 0.004), confirming the stronger cueing effects in American participants observed in previous sections. Consistently, in the bias model (Fig. 6B), we did not find a 3-way interaction (χ^2^(2) = 0.33, *p* = 0.85) or any other interaction driven by the stimulus race (*p*s > 0.76). Instead, we observed a significant main effect of culture (χ^2^(1) = 5.69, *p* = 0.02) and a significant interaction between culture and attention cues (χ^2^(2) = 8.04, *p* = 0.02). Therefore, the stronger cueing effects observed in American participants were consistent, regardless of the race of the face stimuli, suggesting that the locally biased visual processing style in American participants was evident in both Korean and American face stimuli during ensemble perception.

**Table 5:** Precision and bias model comparison results.

Effects	χ^2^
Precision (regression slope)	
Attentional cue	0.70
Culture	13.14***
Stimulus race	28.10***
Attention cue × culture	8.94*
Attention cue × stimulus race	0.77
Culture × stimulus race	0.99
Attention cue × culture × stimulus race	0.40
Bias (decision boundary)	
Attention cue	5.49
Culture	5.69*
Stimulus race	1.14
Attentional cue × culture	8.04*
Attentional cue × stimulus race	0.01
Culture × stimulus race	0.05
Attention cue × culture × stimulus race	0.33

***p < 0.001, **p < 0.01, *p < 0.05.

## General discussion

The current study investigated how the weighted averaging process during face ensemble perception modulated by observers’ different cultural backgrounds via exogenous cueing. We hypothesized that American participants, who tended to be more tuned to local visual information than Korean participants, would be more strongly affected by an attention cue toward an individual face when perceiving the average emotion of a group of faces. In Experiment 1, we first tested this hypothesis using East Asian facial stimuli and observed that American participants were more strongly affected by attention cues than were Korean participants during face ensemble perception. In Experiment 2, we used Caucasian facial stimuli to evaluate whether the familiarity of faces was the main factor in the findings of Experiment 1. We again observed that American participants were more strongly influenced by the attention cue than Korean participants, even when judging the average emotion of a group of Caucasian faces that were more familiar to them. These results support our hypothesis that American participants with a locally biased visual processing style are more likely to weigh an attended face when perceiving an ensemble of faces, regardless of the race of the faces with which they interact.

### Cultural differences in weighted averaging

The different weighted averaging in the two cultural groups provide new insights into how the dominant default visual processing style in each cultural group operates during ensemble face perception. Previous studies^4,18,19^ have shown that Easterners have better face ensemble perception than Westerners because of their higher sensitivity to global information. However, it remains unknown how such cultural differences in global visual processing would interact with the unavoidable interference by local information, strengthened by spatial attention. Our study suggests that when attention cues selectively prioritize local information during ensemble perception, Easterners and Westerners differentially react to local information. Specifically, we showed that American participants who are more inclined to process local visual information tend to make judgments about global information under the impact of local information that captures spatial attention. On the other hand, we have shown that Korean participants are less affected by the attention cue, and their ensemble processing performance is relatively unbiased by the attention cue, despite its saliency. To the best of our knowledge, this study provides the first evidence that people with different cultural backgrounds differently weigh local visual information when extracting gist from the social environment.

Both the precision and biases of ensemble perception contributed to the stronger weighted averaging in the American participants than in the Korean participants. Particularly in Experiment 1, we observed that American participants perceived the overall mood of a face array less precisely during ensemble perception and tended to be more biased toward the attended local face than toward the unattended faces. These two patterns were observed because American participants strongly relied on cued faces while judging the overall mood of the group. If they did not have accurate information about surrounding faces other than the cued one, the participants maximized the attentional weights on the cued face but minimized the weight on the other faces, reflected in the lower slopes of linear functions and shifts of decision boundaries, compared to Korean participants. Thus, the response precision and bias results provide an elaborate explanation of how Americans were more strongly affected by attention cues than Koreans were during ensemble perception.

One interesting aspect of our data is the discrepancy in the number of excluded participants between the two culture groups (Experiment 1: four Koreans vs. 14 Americans; Experiment 2: three Koreans vs. five Americans). One might argue from such a pattern that those American participants may have misunderstood the nature of the ensemble task and responded solely according to the cued faces while ignoring the other faces. In fact, some participants did show a response pattern seemingly indicating that they had responded according to the cued faces, e.g., flat slopes fixed at high/low *p*(“positive response”) in the HC/AC (see participants 82, 87, 90, 99, and 105 in Supplementary Fig. 1).

However, this scenario is less likely because the experimental settings and instructions were carefully matched (judge the ‘overall’ mood of the face arrays) among all participant groups. Instead, we consider that those participants were extremely influenced by attention cues. Given that Americans rely more on local information by default in their daily visual experiences, observing more American participants being extremely influenced by local cues is not surprising. Nevertheless, because we were interested in investigating the ‘weighted average,’ not just the cueing effects, we excluded those participants who might have solely relied on the cued faces during ensemble judgments. Even after excluding those, our results still indicated that Americans weighed attended faces more than Koreans.

### Other-race effects (ORE) during weighted average process

The cueing effect represented by the regression slopes and decision boundaries was similar between Experiments 1 and 2 when directly compared in a combined statistical model (Fig. 6). However, when we conducted a separate analysis in each experiment, the slope-driven cueing effect in Americans was only observed in Experiment 1 but not in Experiment 2. This might imply that there was a slight difference in participants’ cognitive processes depending on the race of the face stimuli and their cultural background, although the observed pattern of distinct cueing effects between cultures was not restricted to a specific race. A potential reason for this subtle statistical difference in regression slopes from the ORE is that people experience processing deficiency with faces from other races but benefit from their own races. According to the literature, one of the factors that drive ORE is the perceptual efficiency of a race in which observers interact more^45–47^. Specifically, frequent exposure and interaction with one’s own race lead to a qualitatively different perceptual style for the faces of that race, shifting from a feature-based process to a configural one^45–47^ (but also see^48^). This suggests that people can encode a same-race face, which is probably encountered the most in their culture, better without separately processing each facial feature (e.g., eyes, nose, and lips) but by holistically comprehending the core information of the face, such as emotion, gender, or identity, in an efficient way. According to a recent finding in ensemble perception^49^ refined representations of individual faces improve ensemble perception owing to reduced noise in individual representations. In parallel with this finding, refined encoding of Caucasian faces, especially by American participants, would improve the quality of the perceived ensemble built upon refined individual faces. Moreover, more accurate representations of unattended faces would help Americans to rely less strongly on attended faces during ensemble processing. In this way, American participants could perceive the ensemble of Caucasian faces more precisely while relying less on the cued face during ensemble perception.

A critical limitation of our study is the missing race information in American participants due to the technical issue, while we tried to recruit non-Asian participants during the data collection. With the absence of objective records on the race information, therefore, we cannot strongly conclude our discussion on the ORE in the previous paragraph. However, according to the previous studies^45–47,50–53^, the ORE has been argued that, rather than the biological race of an observer, the frequency of interactive experiences with any given race determines how fluently the observer can process the faces of that race. Since we strictly screened the Korean participants’ experiences abroad, we think that at least there would be enough differences between the two cultural groups in the amount of interaction with different races. In this sense, American participants who had more interacted with Caucasian faces in their daily lives would have less difficulty processing those faces in Experiment 2, qualitatively supporting our discussion in this section.

### Limitations and future direction

Regarding the sample issue, a replication study with concrete race information will be helpful for drawing a stronger conclusion. Also, our sample comprised only college students. Since children^54^ and older adults^55^ use more distributed attention (analogous to holistic processing style) than young adults, the pattern of results found here might be different. Perhaps American children and older adults might show less attentional cueing effects, similar to young Korean adults. Further studies are necessary to clarify this issue.

Then, it is unclear why the precision of ensemble perception was higher in Experiment 2, even for Korean participants, than in Experiment 1. One potential reason for this is the differences in emotional clarities between the face image sets used in our study (Yonsei Face database^39^ vs. FACES database^44^). Both datasets consisted of six facial expressions (fearful, happy, surprised, sad, disgusted, and angry), and they validated the emotional expressions by having human participants judge the most plausible emotions for individual faces among the six options. According to the results, the accuracies of these judgment experiments were 94.47% for happy faces and 79.59% for angry faces for East Asian faces^39^, and 100% and 100% for Caucasian faces^44^. These scores imply that East Asian face sets may have somewhat less clear emotional expressions than the Caucasian face set, although we cannot directly quantify these differences because the data were collected from different groups of participants. Notably, Easterners tend to judge the emotion intensity lower than Westerners^56^. Thus, in Experiment 2, clearer emotional expressions on Caucasian faces might help both Koreans and Americans to perform ensemble tasks better.

## Conclusions

The present study provides evidence that attentional cueing effects during ensemble perception are modulated by culture and culture-associated information processing styles. Considering this cultural effect, future studies may need to be careful while generalizing their results to the full population without considering the participants’ acquired characteristics, such as their cultural background, or the potential impact of stimuli, such as the ORE.

### Supplementary Information

Supplementary Information.

## Data Availability

The datasets generated and/or analyzed during the current study are available in the Open Science Framework repository, https://osf.io/6w42k/.
