# Supplementary material for: Americans weigh an attended emotion more than Koreans in overall mood judgments
Source: Sci Rep. 2023 Nov 7;13:19323. doi: 10.1038/s41598-023-46723-7 (PMC10630378; doi:10.1038/s41598-023-46723-7)
Supplement: Supplementary file 1 — Supplementary Information. [file 41598_2023_46723_MOESM1_ESM.docx]

**Supplementary Materials**

1. **Individual participant results.**

*Supplementary Figure 1. Individual performance of Korean participants in Experiment 1. Participants 21, 22, 40, and 48 (outlined by dashed lines) were excluded from the final analysis.*

*Supplementary Figure 2. Individual performance of American participants in Experiment 1. Participants 61, 63, 64, 66, 82, 83, 87, 88, 90, 99, 102, 103, 105, and 107 were excluded from the final analysis.*

*Supplementary Figure 3. Individual performance of Korean participants in Experiment 2. Participants 130, 137, and 143 were excluded from the final analysis.*

*Supplementary Figure 4. Individual performance of American participants in Experiment 2. Participants 172, 174, 176, 184, and 203 were excluded from the final analysis.*

1. **Summary tables of multi-level logistic regressions**

*Supplementary Table 1. Multi-level regression results of Experiment 1.*

| Effect | Estimate | *SE* | 95% CI [*LL, UL*] | *p*-value |
| --- | --- | --- | --- | --- |
| Intercept | 0.238 | 0.049 | [0.14, 0.33] | < 0.001 *** |
| Positive valence | 0.542 | 0.014 | [0.51, 0.57] | < 0.001 *** |
| AC | -0.203 | 0.055 | [-0.31, -0.10] | < 0.001 *** |
| HC | 0.045 | 0.054 | [-0.06, 0.15] | 0.40 |
| American | -0.076 | 0.071 | [-0.21, 0.06] | 0.28 |
| Positive valence x AC | -0.017 | 0.020 | [-0.06, 0.02] | 0.38 |
| Positive valence x HC | -0.014 | 0.020 | [-0.05, 0.03] | 0.47 |
| Positive valence x American | -0.032 | 0.020 | [-0.07,0.01] | 0.11 |
| AC x American | -0.083 | 0.079 | [-0.24, 0.07] | 0.29 |
| HC x American | 0.138 | 0.077 | [-0.01, 0.29] | 0.07 |
| Positive valence ratio x AC x American | -0.097 | 0.028 | [-0.15, -0.04] | < 0.001 *** |
| Positive valence ratio x HC x American | -0.048 | 0.029 | [-0.10, 0.01] | 0.09 |

*Note. NC is the baseline condition for Attention cue (AC & HC) and its interactions. Korean was the base line condition for Culture (American) and its interactions. Estimates are presented in Log Odds ratio scale. Some very small values were rounded to three decimal places instead of two decimal places.*

*Supplementary Table 2. Multi-level logistic regression results of Experiment 2.*

| Effect | Estimate | *SE* | 95% CI [*LL, UL*] | *p*-value |
| --- | --- | --- | --- | --- |
| Intercept | 0.166 | 0.052 | [0.06, 0.27] | = 0.001 ** |
| Positive valence | 0.729 | 0.016 | [0.69, 0.76] | < 0.001 *** |
| AC | -0.239 | 0.071 | [-0.38, -0.10] | < 0.001 *** |
| HC | 0.035 | 0.069 | [-0.10, 0.17] | 0.62 |
| American | -0.065 | 0.074 | [-0.21, 0.08] | 0.38 |
| Positive valence x AC | -0.031 | 0.023 | [-0.07, 0.01] | 0.17 |
| Positive valence x HC | -0.029 | 0.023 | [-0.07, 0.01] | 0.21 |
| Positive valence x American | -0.032 | 0.023 | [-0.13, -0.04] | < 0.001 *** |
| AC x American | -0.084 | 0.101 | [-0.36, 0.03] | 0.10 |
| HC x American | 0.164 | 0.099 | [0.07, 0.45] | 0.008 *** |
| Positive valence ratio x AC x American | -0.042 | 0.032 | [-0.10, 0.02] | 0.19 |
| Positive valence ratio x HC x American | 0.006 | 0.032 | [-0.06, 0.07] | 0.85 |

*Note. NC is the baseline condition for Attention cue (AC & HC) and its interactions. Korean was the base line condition for Culture (American) and its interactions. Estimates are presented in Log Odds ratio scale. Some very small values were rounded to three decimal places instead of two decimal places.*

*Supplementary Table 3. Multi-level linear regression results comparing precision of Experiments 1 & 2.*

| Effect | Estimate | *SE* | 95% CI [*LL, UL*] | *p*-value |
| --- | --- | --- | --- | --- |
| Intercept | 0.558 | 0.025 | [0.51, 0.61] | < 0.001 *** |
| AC | -0.019 | 0.025 | [-0.07, 0.03] | 0.45 |
| HC | 0.017 | 0.025 | [-0.07, 0.03] | 0.49 |
| American (culture) | -0.028 | 0.037 | [-0.10, 0.04] | 0.45 |
| Caucasian (stimulus race) | 0.208 | 0.036 | [0.14, 0.27] | < 0.001 *** |
| AC x American | -0.107 | 0.037 | [-0.18, -0.04] | 0.003 ** |
| HC x American | -0.053 | 0.037 | [-0.12, 0.02] | 0.14 |
| AC x Caucasian | -0.015 | 0.035 | [-0.08, 0.05] | 0.67 |
| HC x Caucasian | -0.025 | 0.035 | [-0.09, 0.04] | 0.47 |
| American x Caucasian | -0.053 | 0.052 | [-0.15, 0.05] | 0.31 |
| AC x American x Caucasian | 0.053 | 0.050 | [-0.05, 0.15] | 0.29 |
| HC x American x Caucasian | 0.063 | 0.050 | [-0.04, 0.16] | 0.21 |

*Note. NC is the baseline condition for Attention cue (AC & HC) and its interactions. Korean was the base line condition for Culture (American) and its interactions. East Asian is the baseline condition for Stimulus race (Caucasian). Some very small values were rounded to three decimal places instead of two decimal places.*

*Supplementary Table 4. Multi-level linear regression results comparing bias of Experiments 1 & 2.*

| Effect | Estimate | *SE* | 95% CI [*LL, UL*] | *p*-value |
| --- | --- | --- | --- | --- |
| Intercept | 0.558 | 0.025 | [0.51, 0.61] | < 0.001 *** |
| AC | -0.019 | 0.025 | [-0.07, 0.03] | 0.45 |
| HC | 0.017 | 0.025 | [-0.07, 0.03] | 0.49 |
| American (culture) | -0.028 | 0.037 | [-0.10, 0.04] | 0.45 |
| Caucasian (stimulus race) | 0.208 | 0.036 | [0.14, 0.27] | < 0.001 *** |
| AC x American | -0.107 | 0.037 | [-0.18, -0.04] | 0.003 ** |
| HC x American | -0.053 | 0.037 | [-0.12, 0.02] | 0.14 |
| AC x Caucasian | -0.015 | 0.035 | [-0.08, 0.05] | 0.67 |
| HC x Caucasian | -0.025 | 0.035 | [-0.09, 0.04] | 0.47 |
| American x Caucasian | -0.053 | 0.052 | [-0.15, 0.05] | 0.31 |
| AC x American x Caucasian | 0.053 | 0.050 | [-0.05, 0.15] | 0.29 |
| HC x American x Caucasian | 0.063 | 0.050 | [-0.04, 0.16] | 0.21 |

*Note. NC is the baseline condition for Attention cue (AC & HC) and its interactions. Korean was the base line condition for Culture (American) and its interactions. East Asian is the baseline condition for Stimulus race (Caucasian). Some very small values were rounded to three decimal places instead of two decimal places.*
